# Supplementary material for: MMP-9 as Prognostic Marker for Brain Tumours: A Comparative Study on Serum-Derived Small Extracellular Vesicles
Source: Cancers (Basel). 2023 Jan 24;15(3):712. doi: 10.3390/cancers15030712 (PMC9913777; doi:10.3390/cancers15030712)
Supplement: Supplementary file 1 [file cancers-15-00712-s001.zip › Supplementary Figures for MMP-9 as a prognostic marker for brain tumours-revision.pdf]

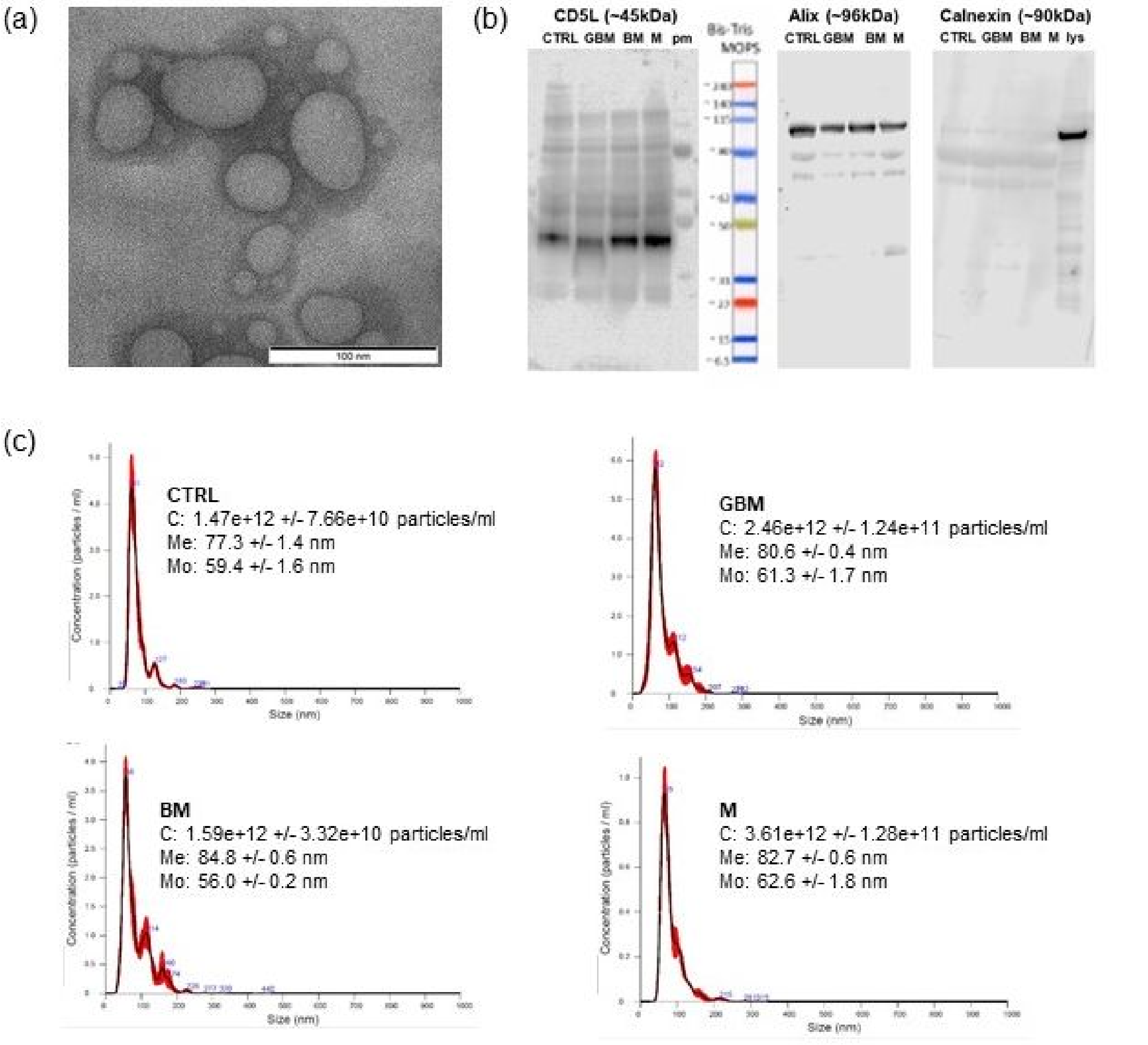

Figure S1. Characterization of serum-isolated particles. (a) Representative transmission electron microscopy (TEM) image of the tested samples with a 100 nm scale bar. (b) Figure shows Alix, CD5L markers, and Calnexin examined by Western blot (WB) analyses. (c) The diagrams display the concentration and size distributions of the serum-derived particles of the four main groups measured by nanoparticle tracking analysis (NTA). (Abbreviations: CTRL, control; GBM, glioblastoma multiforme; BM, brain metastasis; M, meningioma; lys, cell lysate; pm, protein marker; C, particle concentration; Me, mean diameter size; Mo, mode diameter size.)

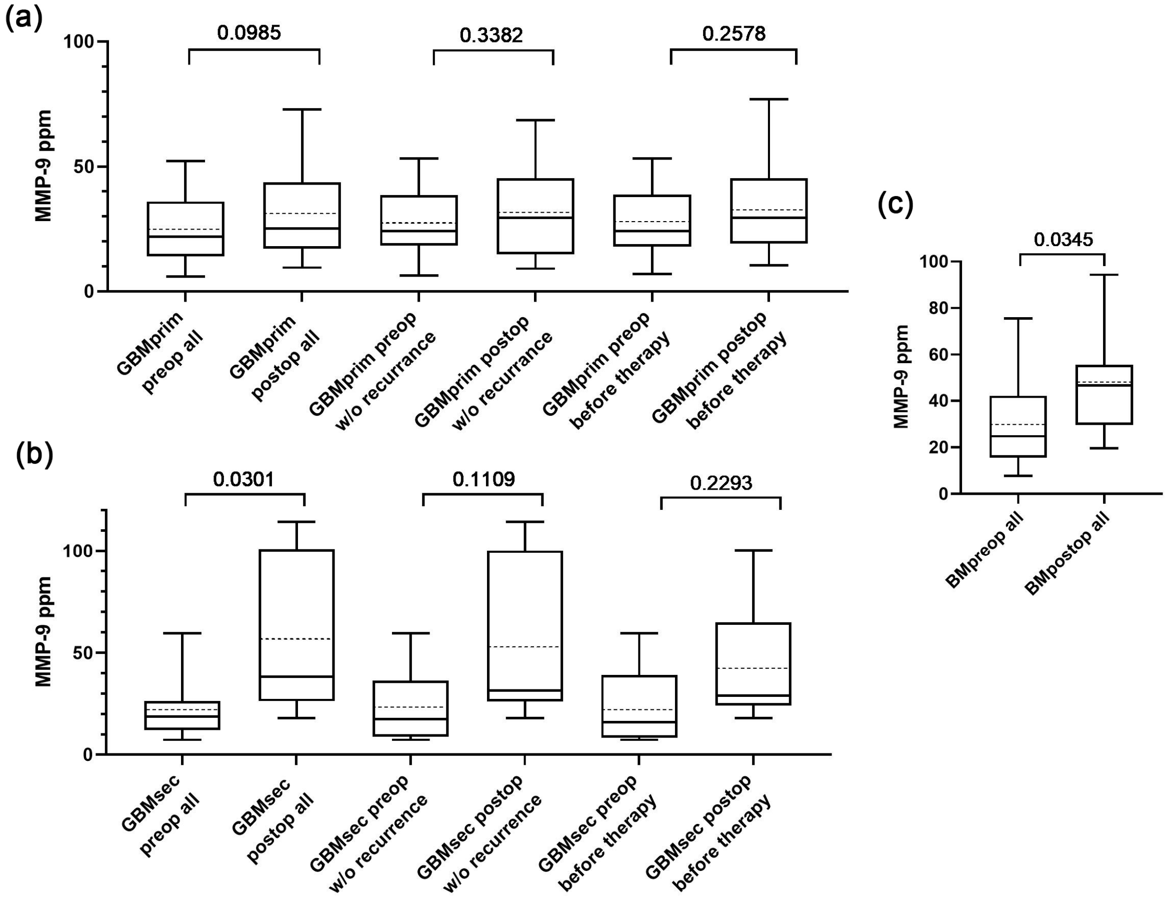

Figure S2. Comparison of all the preoperative and the postoperative GBM and BM samples in terms of MMP-9 level. (**a**) MMP-9 levels of serum derived sEVs in primary GBM group before and after surgical resection (boxplots show median with interquartile range, error bars range from the 5-95 percentiles); nGBMprim preop all = 67 nGBMprim postop all = 32) with the exception of recurrent tumors (nGBMprim preop w/o recurrence = 52, nGBMprim postop w/o recurrence = 23) and those who received therapy (nGBMprim preop before therapy = 52, nGBMprim postop before therapy = 23). (**b**) MMP-9 levels of serum derived sEVs in secondary GBM group before and after surgical resection (nGBMsec preop all = 9 nGBMsec postop all = 9) with the exception of recurrent tumours (nGBMsec preop w/o recurrence = 7, nGBMsec postop w/o recurrence = 8) and those who received therapy (nGBMsec preop before therapy = 6, nGBMsec postop before therapy = 7). (**c**) MMP-9 levels in BM group considering that sampling was made before or after surgical resection (nBMpreop all = 27, nBMpostop all = 10).

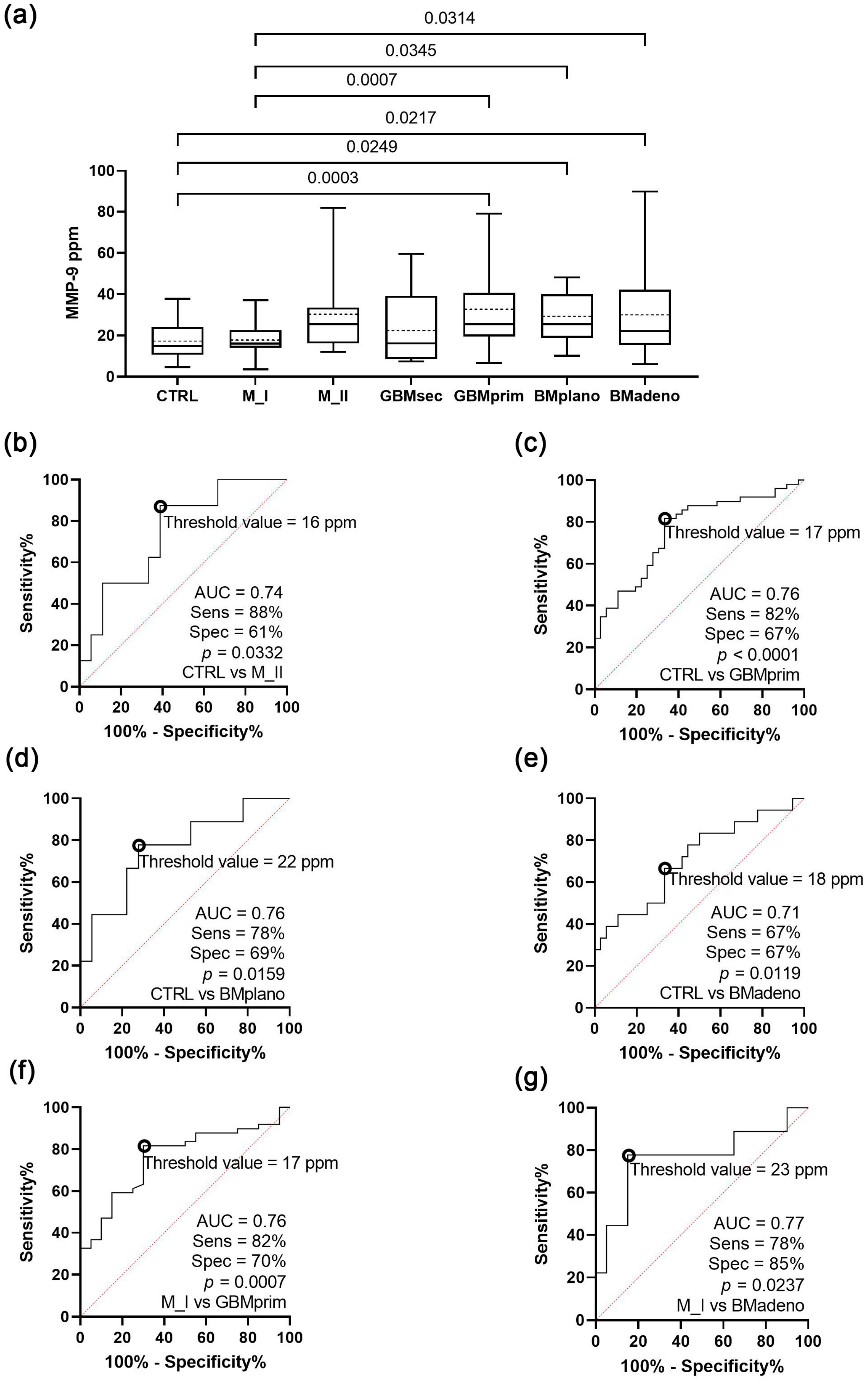
(a)
0.0314
0.0345
0.0007
0.0217
0.0249
0.0003
MMP-9 ppm
100
80
60
40
20
0
CTRL
M_I
M_II
GBMsec
GBMprim
BMplano
BMadeno
(b)
Threshold value = 16 ppm
AUC = 0.74
Sens = 88%
Spec = 61%
p = 0.0332
CTRL vs M_II
Sensitivity%
100% - Specificity%
(c)
Threshold value = 17 ppm
AUC = 0.76
Sens = 82%
Spec = 67%
p < 0.0001
CTRL vs GBMprim
Sensitivity%
100% - Specificity%
(d)
Threshold value = 22 ppm
AUC = 0.76
Sens = 78%
Spec = 69%
p = 0.0159
CTRL vs BMplano
Sensitivity%
100% - Specificity%
(e)
Threshold value = 18 ppm
AUC = 0.71
Sens = 67%
Spec = 67%
p = 0.0119
CTRL vs BMadeno
Sensitivity%
100% - Specificity%
(f)
Threshold value = 17 ppm
AUC = 0.76
Sens = 82%
Spec = 70%
p = 0.0007
M_I vs GBMprim
Sensitivity%
100% - Specificity%
(g)
Threshold value = 23 ppm
AUC = 0.77
Sens = 78%
Spec = 85%
p = 0.0237
M_I vs BMadeno
Sensitivity%
100% - Specificity%

Figure S3. Differences among controls and various CNS tumour patients based on MMP-9 level of sEVs originated from preoperative serum samples. (**a**) Diagram shows the MMP-9 level of serum sEVs among the seven patient groups (boxplots show median with interquartile range, error bars range from the 5-95 percentiles); nCTRL = 36, nM_I = 20, nM_II = 8, nGBMsec = 5, nGBMprim = 49, nBMplano = 9, nBMadeno = 18) (**b**) ROC curves for comparing the patient groups having significant differences in MMP-9 levels (nCTRL = 36, nM_I = 20, nM_II = 8, nGBMsec = 5, nGBMprim = 49, nBMplano = 9, nBMadeno = 18; threshold values of the group-membership score are marked with black circles).
