# Supplementary figures and images for: MMP-9 as Prognostic Marker for Brain Tumours: A Comparative Study on Serum-Derived Small Extracellular Vesicles
Source: Cancers (Basel). 2023 Jan 24;15(3):712. doi: 10.3390/cancers15030712 (PMC9913777; doi:10.3390/cancers15030712)

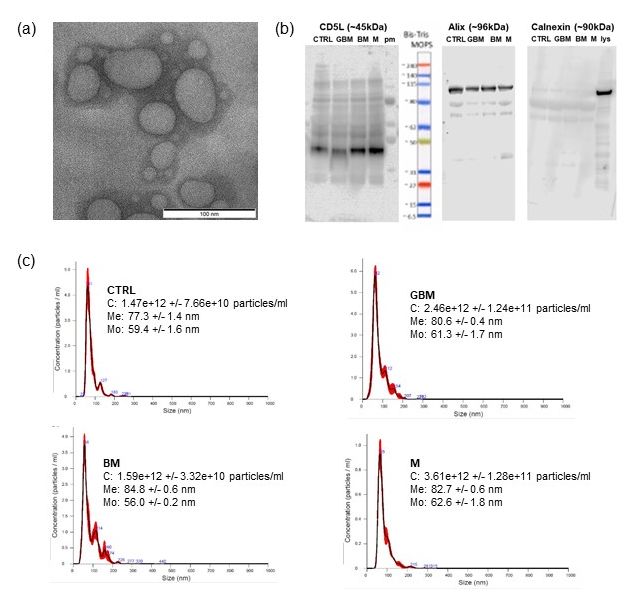

Supplement: Supplementary file 1 [file cancers-15-00712-s001.zip › Figure S1 Characterisation of serum-isolated particles.jpg]

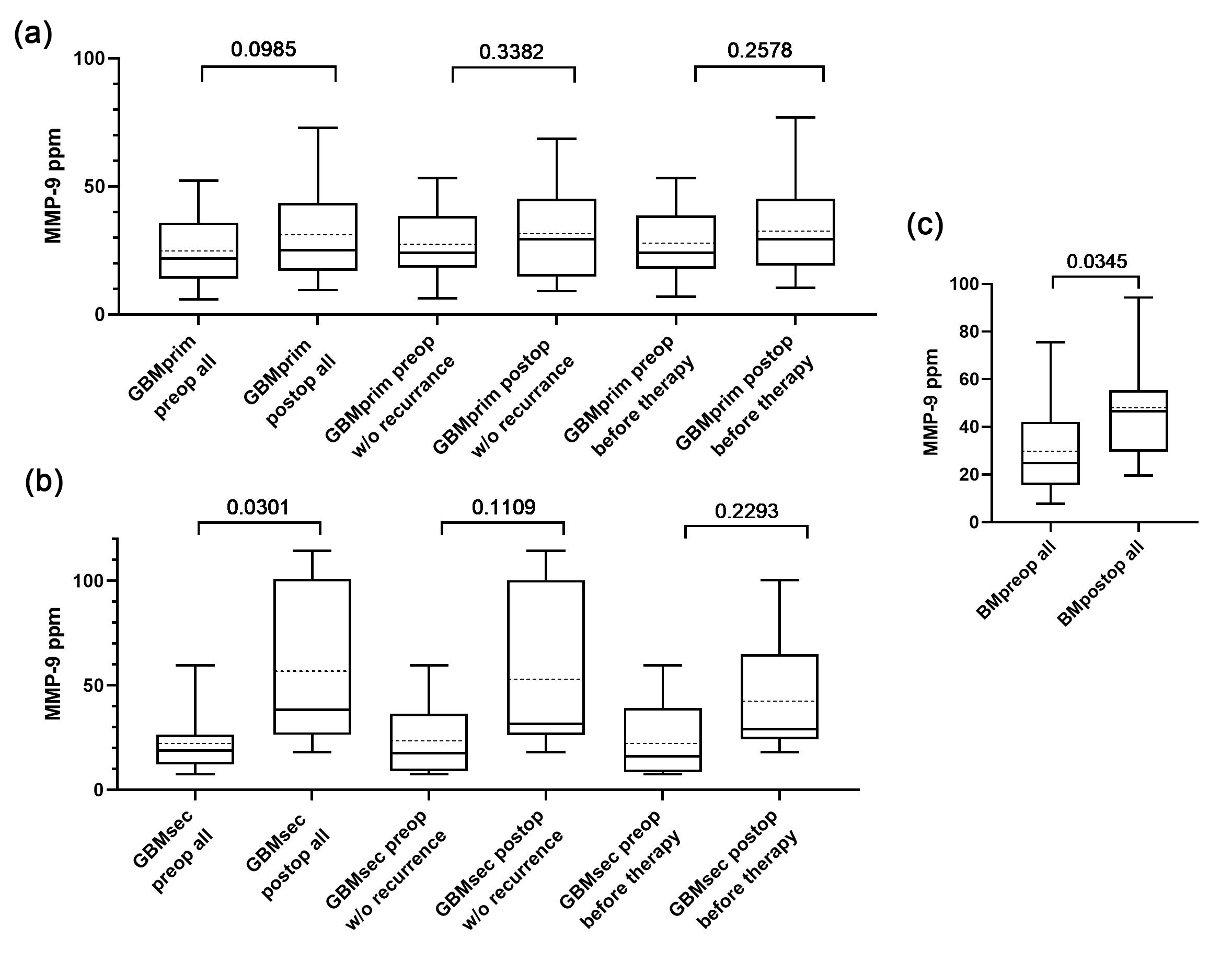

Supplement: Supplementary file 1 [file cancers-15-00712-s001.zip › Figure S2 Comparison of all the preoperative and the postoperative GBM and BM samples in terms of MMP-9 level .jpg]

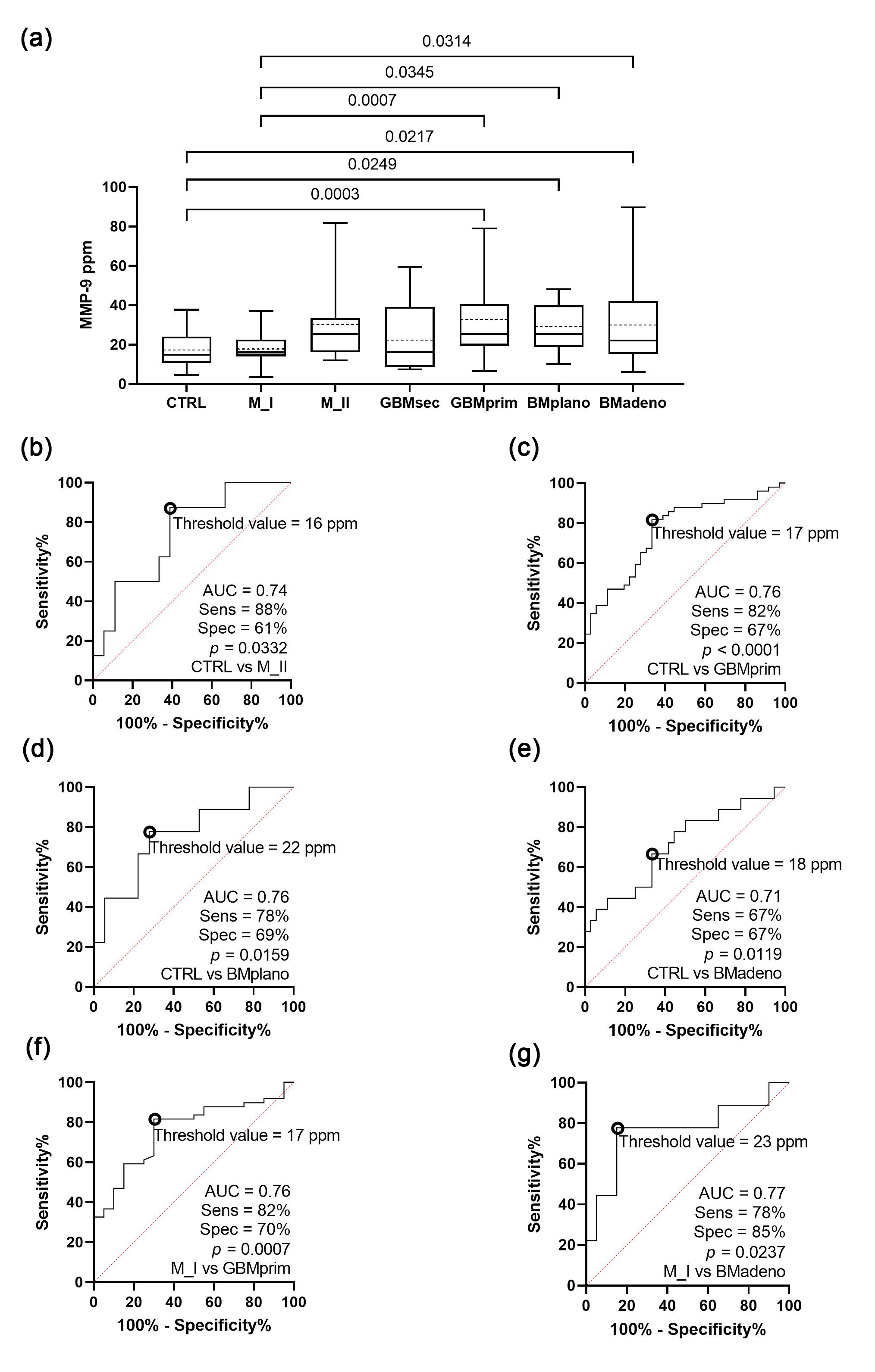

Supplement: Supplementary file 1 [file cancers-15-00712-s001.zip › Figure S3 Differences among controls and various CNS tumour patients based on MMP-9 level of sEVs originated from preoperative serum samples .jpg]
